# The Maryland Acute Stroke Emergency Medical Services Routing Pilot: Expediting Access to Thrombectomy for Stroke

**DOI:** 10.3389/fneur.2021.663472

**Published:** 2021-08-31

**Authors:** Taylor Haight, Burton Tabaac, Kelly-Ann Patrice, Michael S. Phipps, Jaime Butler, Brenda Johnson, Anna Aycock, Linda Toral, Karen L. Yarbrough, Chad Schrier, Erin Lawrence, Adrian Goldszmidt, Elisabeth B. Marsh, Victor C. Urrutia

**Affiliations:** ^1^Department of Neurology, Johns Hopkins University School of Medicine, Baltimore, MD, United States; ^2^Department of Neurology, University of Maryland School of Medicine, Baltimore, MD, United States; ^3^The Johns Hopkins Hospital, Baltimore, MD, United States; ^4^Maryland Institute for Emergency Medical Services System (MIEMSS), Baltimore, MD, United States; ^5^Sinai Hospital, Baltimore, MD, United States; ^6^University of Maryland Medical Center, Baltimore, MD, United States; ^7^Johns Hopkins Bayview Medical Center, Baltimore, MD, United States

**Keywords:** emergency medical services, routing protocol, acute stroke, large vessel occlusion, mechanical thrombectomy, healthcare delivery assessment

## Abstract

**Background:** Mechanical thrombectomy (MT) is the standard of care for acute ischemic stroke caused by large vessel occlusion, but is not available at all stroke centers. Transfers between hospitals lead to treatment delays. Transport directly to a facility capable of MT based on a prehospital stroke severity scale score has been recommended, if transportation time is less than 30 min.

**Aims:** We hypothesized that an Emergency Medical Services (EMS) routing algorithm for stroke, using the Los Angeles Motor Scale (LAMS) in the field, would improve time from last known well to MT, without causing patients to miss the IV Thrombolysis (IVT) window.

**Methods:** An EMS algorithm in the Baltimore metro area using the LAMS was implemented. Patients suspected of having an acute stroke were assessed by EMS using the LAMS. Patients scoring 4 or higher and within 20 h from last known well, were transported directly to a Thrombectomy Center, if transport could be completed within 30 min. The algorithm was evaluated retrospectively with prospectively collected data at the Thrombectomy Centers. The primary outcome variables were proportion of patients with suspected stroke rerouted by EMS, proportion of rerouted ischemic stroke patients receiving MT, time to treatment, and whether the IVT window was missed.

**Results:** A total of 303 patients were rerouted out of 2459 suspected stroke patients over a period of 6 months. Of diverted patients, 47% had acute ischemic stroke. Of these, 48% received an acute stroke treatment: 16.8% IVT, 17.5% MT, and 14% MT+IVT. Thrombectomy occurred 119 min earlier in diverted patients compared to patients transferred from other hospitals (*P* = 0.006). 55.3% of diverted patients undergoing MT and 38.2% of patients transferred from hospital to hospital were independent at 90 days (modified Rankin score 0–2) (*P* = 0.148). No patient missed the time window for IVT due to the extra travel time.

**Conclusions:** In this retrospective analysis of prospectively acquired data, implementation of a pre-hospital clinical screening score to detect patients with suspected acute ischemic stroke due to large vessel occlusion was feasible. Rerouting patients directly to a Thrombectomy Center, based on the EMS algorithm, led to a shorter time to thrombectomy.

## Introduction

The modern treatment of stroke requires organization of multiple systems of care for maximum efficiency, in order to obtain the best outcomes. The efficacy of mechanical thrombectomy (MT) for the treatment of stroke due to large vessel occlusion (LVO) has been well established since 2015, for up to 6 h from last known well (1–5). More recently, the window for treatment with MT has been extended to 24 h from last known well (LKW) in imaging-selected patients (6, 7). Studies have shown that time delays decrease the probability of good outcome with endovascular therapy, with one analysis showing 12–15% relative reduction in likelihood of a good clinical outcome for every 30 min delay in angiographic reperfusion (8–12).

In the United States, a multi-tier stroke certification system exists that generally establishes 4 levels of stroke expertise at hospitals. The terminology varies somewhat according to the certifying organization, generally corresponding to: (1) Acute Stroke Ready Hospital (ASRH) – has proceses and capability in place to acutely evaluate stroke patients and administer thrombolytic therapy, most patients require transfer to higher levels of care. (2) Primary Stroke Center (PSC) – are capable of treating most stroke patients and administering thrombolytic therapy. (3) Thrombectomy-Capable Stroke Center (TSC) – are capable of treating most stroke patients, including thrombolytic therapy administration and mechanical thrombectomy. (4) Comprehensive Stroke Center (CSC) – are capable of treating the most complex stroke patients including thrombolytic therapy administration, mechanical thrombectomy, and comprehensive management of hemorrhagic stroke (13, 14).

It has been recognized that given the limited availability of centers capable of MT (CSC/TSC), EMS routing protocols and processes for quick evaluation and transfer of patients need to be developed and implemented (15, 16). The challenges have been: (1) Timely and accurate recognition of eligible patients in the field and what tool to use, (2) The possibility of missing the IV thrombolysis (IVT) window, (3) concerns with overwhelming TSC and CSCs by using a high sensitivity and low specificity tool, (4) ease of training and implementation by EMS personnel.

In 2017, the American Heart Association's (AHA) Mission: Lifeline, published an algorithm for EMS routing for acute stroke, this algorithm was recently updated to accommodate the inclusion of patients with potential LVO that can be treated up to 24 h from last known well and that transport time can be up to 30 min (17, 18).

Froehler et al. in a study from the STRATIS registry found that transfers between hospitals were associated with significant delays in treatment (19). Additionally, they performed a hypothetical bypass analysis to evaluate the potential impact of EMS routing directly to MT-capable centers. This showed that while mean time to IVT administration was delayed by 12 min, endovascular treatment would be done 91 min sooner if patients were transferred directly to an MT-capable center, as opposed to being transferred from a PSC. Another decision analysis model showed that transport to a thrombectomy-capable center was optimal at longer bypass times as the probability of an LVO increased (20).

In 2016, the Maryland Institute for Emergency Medical Services Systems (MIEMSS) established the Los Angeles Motor Scale (LAMS) as a tool to convey stroke severity and likelihood of LVO. The LAMS is a validated scale that was designed for pre-hospital and ED use, (21) and has been shown to have high sensitivity and specificity for predicting the presence of large artery anterior circulation occlusion (22–24). At the time, while other scales were evaluated, LAMS was selected for its ease of use and training. In 2017, MIEMSS adopted a new acute stroke EMS routing protocol within Baltimore City limits as a 6-month pilot. An observational restrospective study of prospective acquired data during the pilot revealed 45/203 (22%) of eligible patients were rerouted, and of those, 20/45 (44%) had ischemic stroke and half of these had an intervention (IVT, MT or both). There were also 10/45 (22%) patients with intracerebral hemorrhage (ICH) that bypassed PSCs, which typically are transferred from PSCs to CSCs (25).

On July 1, 2019, MIEMSS updated the EMS routing protocol to surrounding counties within a 30 min travel time to a TSC or CSC, and the protocol was changed to include patients with acute stroke within 20 h of LKW that potentially could receive treatment by 24 h.

## Aims

We hypothesized that the implementation of the EMS routing algorithm for stroke, using LAMS in the field, would improve time from last known well to mechanical thrombectomy. We conducted a study to determine the proportion of suspected stroke patients that were rerouted, the proportion of mechanical thrombectomy performed, and the time to treatment including whether it would cause patients to miss the IVT window.

## Methods

Data that support the findings of this study are available from the corresponding author upon reasonable request.

Maryland is a state of 6.046 million people. There are 47 acute care hospitals in the state, of which 45 are Base Stations which is a requirement for specialty hospital designation. Of the Base Station Hospitals, 36 are Primary Stroke Centers (PSC) and 3 are Comprehensive Stroke Centers, designated by MIEMSS. One of the PSCs is a Thrombectomy Stroke Center (TSC), certified by The Joint Commission; Maryland does not have a Thrombectomy Stroke Center certification program.

**Figure 1:**
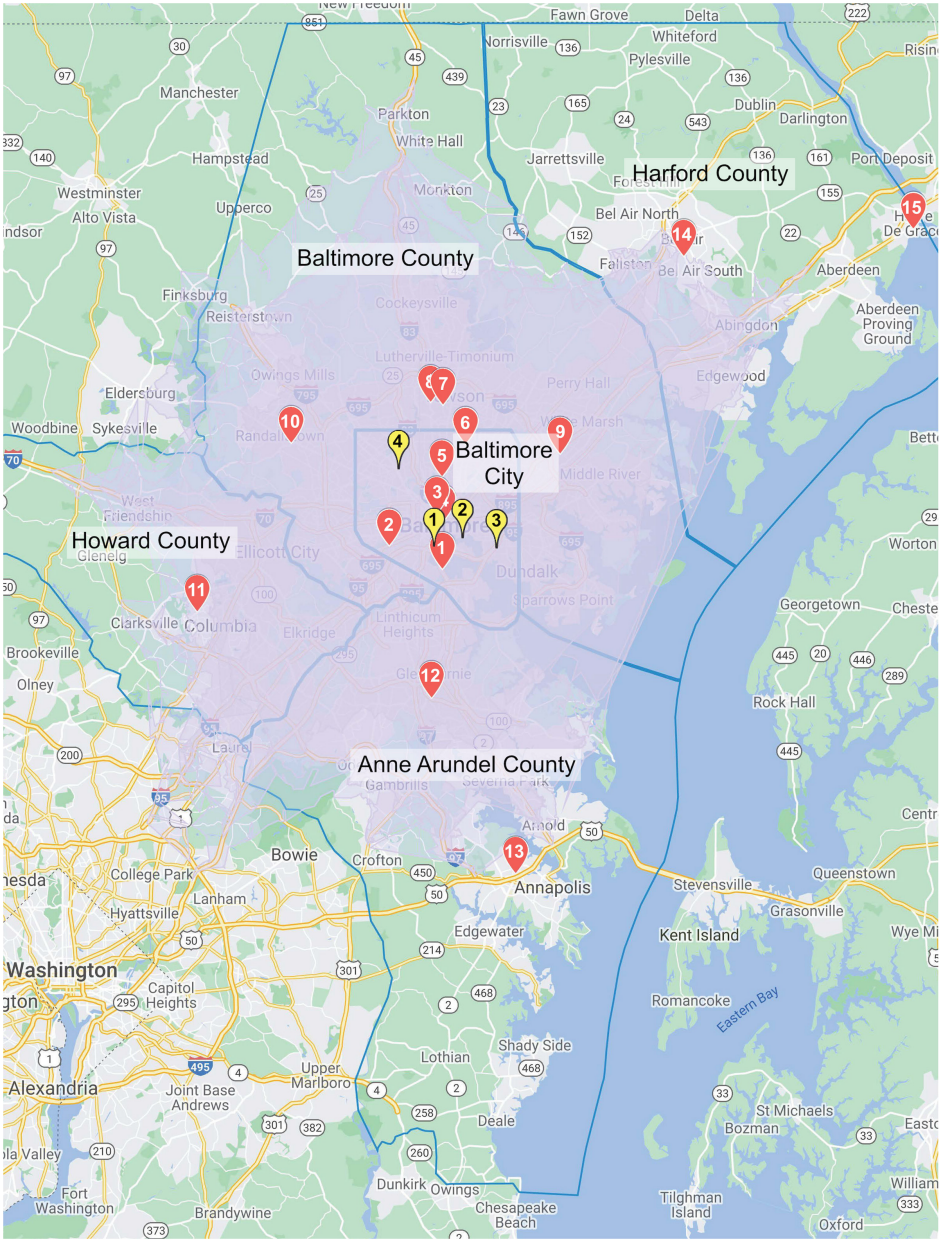
The map depicts the area covered by the re-routing protocol's 30 min drive-time radius. The red markers represent Primary Stroke Centers and the yellow markers represent Thrombectomy Centers.

Within the 30 minute driving time radius from the three CSC and one TSC (Thrombectomy Centers), there are 15 hospitals, not including the 4 Thrombectomy Centers. All 15 hospitals are PSCs. These are located in Baltimore City, Baltimore County, Anne Arundel County, Howard County and Harford County. This region is the most highly populated in Maryland with roughly 3 million inhabitants. Figure 1 represents a map with the PSC hospitals and Thrombectomy Centers, Table 1 includes the distances and approximate driving time between centers.

**Table 1:** Drive time from PSC to Thrombectomy Centers (TC).

	**TC #2**		**TC #3**		**TC #1**		**TC #4**	
**Anne arundel county**	**Minutes**	**Miles**	**Minutes**	**Miles**	**Minutes**	**Miles**	**Minutes**	**Miles**
PSC #13	41	30	38	29.4	32	28	47	35.7
PSC #12	27	17.8	20	15.1	20	13.8	34	21.5
**Baltimore county**								
PSC #9	20	8.4	13	8	21	14.1	24	19
PSC #10	28	18.1	28	21.8	21	16.3	20	8
PCS #7	21	10	22	16.4	22	9.8	13	4.7
PSC # 8	21	10.8	22	17	23	8.3	13	5
**Baltimore city**								
PSC #3	9	2	18	4.6	4	1.3	13	6
PSC #5	13	3.5	18	6.7	12	3.5	12	5.7
PSC #6	16	5.1	17	6.8	19	6.5	14	4.6
PSC #4	6	1.5	16	4.3	8	1.7	12	6.7
PSC #1	14	5.3	15	6.2	8	3.5	21	10.9
PCS #2	15	6.4	16	8.4	9	4.6	22	12
**Harford County**								
PSC #15	45	35.2	37	32.8	45	40.3	50	43.5
PSC #14	34	26.1	27	23.6	35	30.1	39	34.4
**Howard County**								
PSC #11	30	23.9	31	26.3	25	22.1	33	21.4

**Figure 2:**
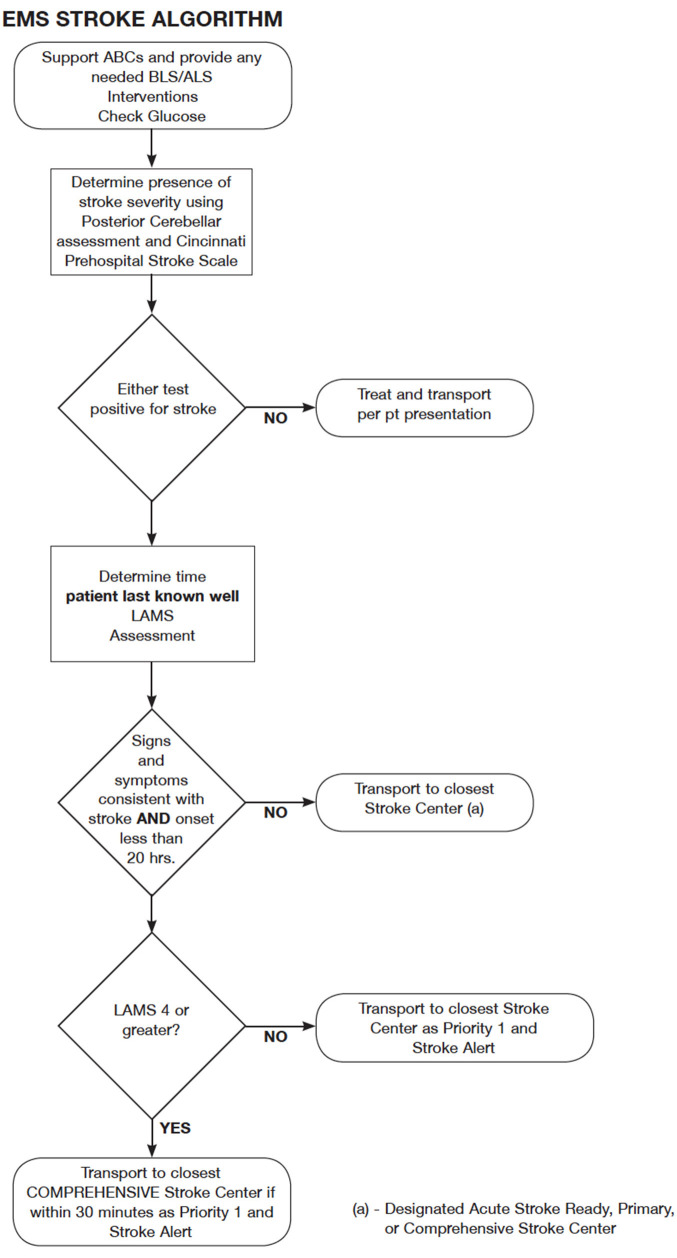
MIEMSS 2019 Acute Stroke EMS Routing Pilot algorithm (with permission from MIEMSS).

The authors of this paper are members of the Maryland Stroke Quality Improvement Committee which works under MIEMSS on quality improvement of stroke care in the state of Maryland. Taking into account recommendations from the authors and available evidence, MIEMSS updated the acute stroke EMS routing protocol in July of 2019 (Figure 2), the rerouting element of the protocol was established as a pilot. The authors designed a retrospective observational study of prospectively collected data from October 1st 2019 to March 31st 2020. This study was approved by the IRB as an expedited review and a waiver of consent was granted based on the following criteria: 1. the research involves no more than minimal risk to subjects; 2. the waiver will not adversely affect the rights and welfare of the subjects; 3. the research could not be practicably carried out without the waiver; and 4. the IRB will advise if it is appropriate for participants to be provided with additional pertinent information after participation. Other participating CSC and TSC's IRB's and MIEMSS agreed to rely on the IRB approval.

EMS personnel were trained via an educational video, explaining the new protocol and the planned study. The protocol was included in The Maryland Protocols for Emergency Medical Services and distributed to all Maryland Base Stations (26).

### Data Collection

Data collected for this study was obtained retrospectively from prospectively collected data for the Thrombectomy Centers' quality improvement (QI) databases, which are required for certification and include modified Rankin score (mRS) at 90 days. Other sources included MEIMSS EMS data, and run sheets of patients brought to the Thrombectomy Centers. A HIPAA (Health Insurance Portability and Accountability Act) waiver was obtained to collect data not normally part of the standard required QI process.

These sources yielded three data sets: (1) suspected stroke patients rerouted to the Thrombectomy centers, (2) suspected stroke patients treated with IVT and/or MT, directly brought to the Thrombectomy Centers, and (3) stroke patients transferred to Thrombectomy Centers for mechanical thrombectomy from the 15 PSCs located within the 30 min drive-time radius. The total number of suspected strokes transported by EMS within the participating Counties was provided by MIEMSS.

We defined a “rerouted patient” in two ways: (1) a patient in which the EMS run sheet was marked as “rerouted,” per protocol, (2) a patient arriving to the Thrombectomy Centers via EMS, and having a PSC that was closer to the location of origin and thus bypassed. The goal was to capture every rerouted patient even in cases of missing or incomplete documentation in the run sheets.

Primary outcome variables included number of patients rerouted to a Thrombectomy Center, proportion of patients receiving mechanical thrombectomy for large vessel occlusion which included ICA, M1, M2 and Basilar artery [MT candidates were selected according to each center's acute stroke treatment protocols, based on the AHA guidelines (15)], time from LKW to IVT or MT, and wether any rerouted patient missed the IVT time window due to rerouting.

### Statistical Analyses

Statistical analysis was descriptive of frequencies and time by calculating the mean and median as well as interquartile and ranges. The times from last known well (LKW) to IVT bolus and to mechanical thrombectomy (MT) were compared between (1) rerouted and transferred patients, and (2) rerouted and directly admitted patients by the Mann-Whitney *U* test, as distributions were not normal. Demographics including gender and race, the outcomes of modified Rankin Scale (mRS) and symptomatic intracerebral hemorrhage (sICH), and the initial NIHSS > 10, between: (1) rerouted and transferred patients and (2) rerouted and direct to ED patients were compared by chi-square. Age was analyzed using *t-*test to compare means.

## Results

### General

A total of 303 out of 2,459 suspected stroke patients were rerouted within Baltimore City and surrounding counties between October 1, 2019 and March 31, 2020. General carachteristics and demographics of rerouted patients are in Table 2. Due to the design of this study, we were not able to ascertain what proportion of the total number of suspected stroke patients were outside of the re-routing protocol radius or met criteria but were misrouted. Rerouted patients represented 33% (303/927) of the total patients brought by EMS to Thrombectomy Centers with a presumptive diagnosis of stroke. 224/303 (74%) of patients rerouted had a LAMS of 4 or 5. Fourty seven percent (143/303) of rerouted patients had a diagnosis of acute ischemic stroke, 8/303 (3%) had TIA, 45/303 (15%) had intracerebral hemorrhage, and 107/303 (35%) had a mimic (Table 3). Among the mimics, the most common diagnoses were mental status change 25/107 (23%), seizure 20/107 (19%), medication/intoxication 18/107 (17%) and hypertensive urgency 17/107 (16%). Only 10/303 (3%) of rerouted patients were transported for more than 30 min, 166/303 (55%) arrived at the Thrombectomy Centers within 16–30 min, and 126/303 (42%) within 15 min. In one patient, transport time was not documented.

**Table 2:** Baseline demographics of rerouted patients.

**Patient characteristic**		
**Sex**		
	Female, *n* (%)	173 (57)
	Male, *n* (%)	130 (43)
Race		
	Black/African American, *n* (%)	107 (35.3)
	White, *n* (%)	180 (59.4)
	Asian, *n* (%)	5 (1.7)
	Hispanic, *n* (%)	1 (0.3)
	American Indian, *n* (%)	1 (0.3)
	Other, *n* (%)	4 (1.3)
	Unknown, *n* (%)	5 (1.7)
Age		
	Median age, years	71.0
	18–29 years, *n* (%)	3 (1.0)
	30–49 years, *n* (%)	25 (8.3)
	50–64 years, *n* (%)	77 (25.4)
	65–79, *n* (%)	118 (38.9)
	80+ years, *n* (%)	80 (26.4)
NIHSS		
	Median NIHSS (IQR)	10 (6–17)
	0–5, *n* (%)	68 (22)
	6–10, *n* (%)	75 (25)
	11–20, *n* (%)	87 (29)
	21–25, *n* (%)	34 (11)
	>25, *n* (%)	9 (3)
	ND, *n* (%)	30 (10)
LAMS		
	Median LAMS (IQR)	4 (3–5)
	0, *n* (%)	26 (9%)
	1, *n* (%)	17 (6%)
	2, *n* (%)	15 (5%)
	3, *n* (%)	20 (7%)
	4, *n* (%)	85 (28%)
	5, *n* (%)	139 (46%)
	ND, *n* (%)	1 (0.3%)

**Table 3:** Final diagnoses of rerouted patients.

**Diagnosis**	***n* (%)**
Ischemic stroke MT only	25 (8)
Ischemic stroke MT+IVT	20 (7)
Ischemic stroke IVT only	24 (8)
Ischemic stroke without intervention, or TIA	82 (27)
ICH or SAH	45 (15)
Stroke mimic	107 (35)
Total	303 (100)

### Ischemic Stroke Population

Ischemic stroke patients were 143 of the 303 rerouted (47%), of these, 48.2% (69/143) had an intervention (IVT or MT or MT+IVT). Of the rerouted ischemic stroke patients that arrived to the Thrombectomy Centers within 5.5 h from LKW, we evaluated if patients that arrived after 4.5 h could have received IVT at a PSC if they had been routed there directly. We found that one patient arrived past the 4.5 h time window for IVT, however they would have arrived to the PSC at 4 h and 20 min, with only 10 min to receive IVT. There was another patient that arrived to the Thrombectomy Center at 4 h and 15 min from LKW and was not treated. The PSC closest to the patient was only 3 min closer, so the patient would have arrived to the PSC with only 18 min to receive IVT. Therefore we conclude that no patients missed the IVT window due to rerouting.

The ambulance was en route within 3.5 h of LKW for 75/143 (52%) of ischemic stroke patients, and none of these patients missed the time window for IVT. In this group, 32% (24/75) received IVT, 27% (20/75) received IVT and MT, and 13% (10/75) underwent MT only, while only 28% (21/75) were ineligible for any intervention for reasons other than time. Fifty-five percent (78/143) of patients with ischemic stroke arrived within the 4.5 h of LKW, and of these, 71% (55/78) underwent an intervention, compared with 40% (4/10) in the 4.5 to 6 h window and 19% (10/52) in the 6 to 24 h window. Three patients out of the 143, had unknown LKW.

The patient demographics, times to treatments and outcomes of ischemic stroke patients undergoing MT and IVT administration by mode of arrival (rerouted, transferred, or direct to ED) are included in Tables 4, 5.

**Table 4:** Demographics and outcomes of MT and MT+IVT by mode of arrival (reroute, transfer, or direct to ED).

	**MT +/− IVT Reroute (*n* = 45)**	**MT +/− IVT Transfer (*n* = 39)**	**MT +/− IVT Direct (*n* = 54)**	**Reroute vs. Transfer *p*-value**	**Reroute vs. Direct *p*-value**
**Age, yrs (SD)**	68.6 (12.7)	65.6 (12.8)	66.5 (14.5)	0.29	0.449
**Gender, F (%)**	24 (53.3)	21 (53.8)	28 (51.9)	0.963	0.883
**Race**					
**White**, ***n*** **(%)**	27 (60.0)	20 (51.3)	20 (37.0)	0.656	**0.026**
**Black**, ***n*** **(%)**	15 (33.3)	13 (33.3)	33 (61.1)		
**Asian**, ***n*** **(%)**	1 (2.2)	1 (2.6)	0 (0)		
**Hispanic**, ***n*** **(%)**	0 (0)	1(2.6)	0 (0)		
**Unknown**, ***n*** **(%)**	2 (4.4)	4 (10.3)	0 (0)		
**Other**, ***n*** **(%)**			1 (1.9)		
**Initial NIHSS>10**, ***n*****(%)**	38 (84.4)	28 (71.8)	39 (72.2)	0.159	0.145
**LKW to Puncture, Median time (min)**	255	374	284	**0.006**	0.489
**mRS 0-2**, ***n*****(%)**	21 (55.3)	13 (38.2)	18 (38.3)	0.148	0.119
**mRS 5-6**, ***n*****(%)**	12 (31.6)	8 (23.5)	11 (23.4)	0.446	0.399
**sICH**, ***n*****(%)**	1 (2.2)	2 (5.1)	3 (5.6)	0.474	0.402

*Bolded p values denote statistical significant result*.

**Table 5:** Demographics and outcomes of IVT by mode of arrival (reroute vs. direct to ED).

	**IVT Reroute (*n* = 24)**	**IVT Direct (*n* = 46)**	***p*-value**
**Age, yrs (SD)**	69.3 (16.3)	69.0 (13.9)	0.947
**Gender, F (%)**	13 (54.2)	21 (45.7)	0.499
**Race**			
**White**, ***n*** **(%)**	13 (54.2)	20 (43.5)	0.344
**Black**, ***n*** **(%)**	10 (41.7)	25 (54.3)	
**Asian**, ***n*** **(%)**	0	0	
**Hispanic**, ***n*** **(%)**	0	0	
**Unknown**, ***n*** **(%)**	0	0	
**Other**, ***n*** **(%)**	1 (4.2)	1 (2.2)	
**Initial NIHSS>10**, ***n*****(%)**	12 (50)	15 (32.6)	0.156
**LKW to Bolus, Median time (min) for all IVT (n includes IVT** **+** **IVT/MT)** ^‡^	124^*^	130^†^	0.987
**mRS 0-2**, ***n*****(%)**	13 (59.1)	29 (69)	0.426
**mRS 5-6**, ***n*****(%)**	4 (18.2)	7 (16.7)	0.879
**sICH**, ***n*****(%)**	1 (4.2)	1 (2.2)	0.635

‡ *For median time to bolus, includes IVT + IVT/MT*.

* *n = 44*,

† *n = 73*.

Comparisons between patients treated with IVT (IVT and IVT + MT) revealed no significant difference in median LKW to bolus. Symptomatic intracerebral hemorrhage (sICH) was 4.2% in rerouted and 2.2% in direct to ED patients (IVT only). Initial NIHSS > 10 for IVT only patients was 50% for rerouted and 32.6% for direct to ED patients. mRS 0–2 at 90 days was the outcome for 59.1% for rerouted and 69% for direct to ED patients.

Sixty one rerouted patients had LVO, of these 45 underwent MT and 16 did not. The reasons for not pursuing MT were more commonly poor baseline (mRS > 1) 6/16 (37.5%), and completed stroke/large core 5/16 (31.2%). There was one patient who was comfort measures only; and 2/16 (12.5%) recanalized with IVT, and 2/16 (12.5%) had low NIHSS. These patients had mostly M1 occlusions 12/16 (75%).

We compared rerouted patients receiving MT with transferred patients treated with MT and direct to ED patients receiving MT. We evaluated the reasons why transferred patients were not rerouted. We found that 14/39 (36%) were outside of the 30 min radius and therefore did not meet criteria for the new algorithm, 7/39 (18%) had LAMS < 4, 5/39 (13%) did not have LAMS documented by EMS, 7/39 (18%) were miscategorized as they met criteria for rerouting, and in 6/39 (15%) documentation was missing, perhaps due to patients arriving by means other than EMS (we could not determine eligibility).

The majority (54%) either were outside of the 30 min radius or did not meet LAMS criteria. Of the rest, 18% were misrouted based on the algorithm and in 28%, we did not have enough information to make a determination.

Among patients who underwent mechanical thrombectomy, those who had been rerouted to the Thrombectomy Centers were treated with a median time 119 min faster than patients who were transferred from another facility [median time from LKW to groin puncture 255 min [interquartile range (IQR) 172 to 466 min) vs. 374 min (IQR 263 to 643 min), *p* = 0.006].

**Figure 3:**
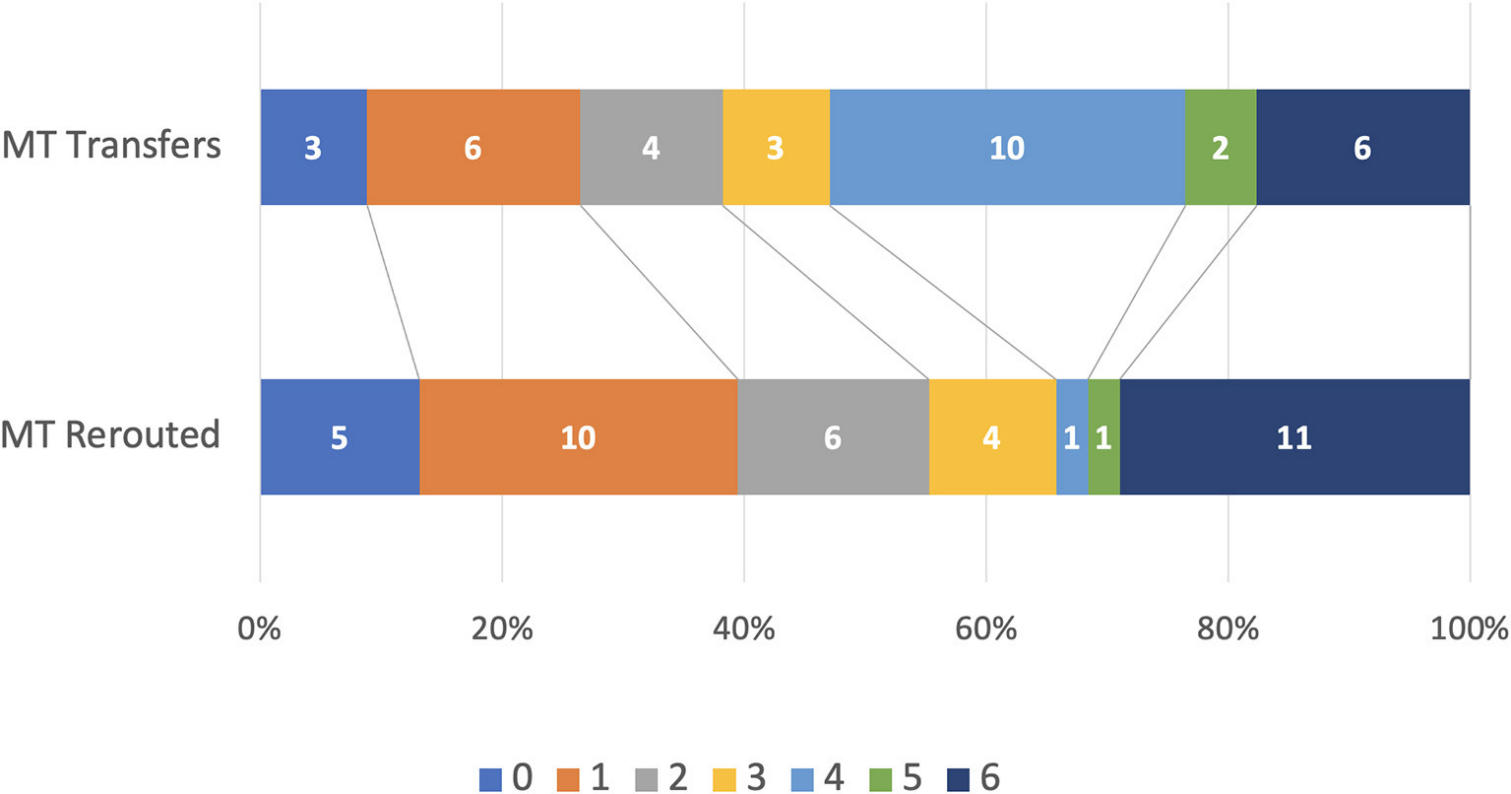
Modified Rankin Score distribution in rerouted vs. transferred MT patients.

We found no statistical difference in time from LKW to groin puncture between patients that were rerouted compared with patients brought directly to the ED. In rerouted patients undergoing MT sICH occurred in 2.2%, and in transferred patients and direct to ED patients undergoing MT it was 5.1% and 5.6% respectively. The mRS 0–2 at 90 days was 55.3% for MT rerouted and 38.2% for MT transferred (Figure 3), and 38.3% for MT direct to ED. Initial NIHSS > 10 was 84.4% for MT rerouted and 71.8 and 72.2% for MT transfers and direct to ED respectively.

LVO distribution was similar in all treated groups. For the MT rerouted patients 18% were ICA, 64% M1, 13% M2, and 4% other, no basilars. For transferred patients there were 31% ICA, 54% M1, 5% M2, and 15% basilar. Direct to the ED MT patients had 26% ICA, 56% M1, 11% M2, and 7% basilar.

## Discussion

The results of this study show that implementation of an EMS routing protocol using an EMS-administered clinical scale is feasible. Allowing for 30 min travel to Thrombectomy Centers did not adversely affect outcomes and patients did not miss the IVT window, and rerouted patients received mechanical thrombectomy with a significantly shorter LKW to groin puncture time.

It is feasible to implement a protocol for selecting patients with likely acute ischemic stroke and LVO in the field, by an EMS-administered clinical scale, and reroute them to a Thrombectomy Center for possible mechanical thrombectomy. Our observational retrospective study of a prospectively implemented EMS routing protocol with prospectively collected data, revealed results that are consistent with what previous modeling studies have suggested, in terms of the decrease in LKW to groin puncture times (27).

Accurate identification of patients with an LVO is critical for an EMS routing protocol. Appropriate patient selection is key for maximizing benefit from MT, as well as preserving limited resources at the Thrombectomy Center for appropriate patients. In our study we did not directly evaluate how many patients, not meeting the LAMS criteria for rerouting, were taken to a PSC and then transferred to a Thrombectomy Center secondarily due to an LVO. However, analyzing the transferred patients we found 18% did not meet LAMS criteria and were found to have LVO and transferred for MT, and 18% met criteria but had been misrouted.

The LAMS was adopted by MIEMSS, based on studies published at the time, showing good performance for predicting anterior LVO in the field, and ease of training and use compared to other similar scales (21–23). The MIEMSS protocol also includes an assessment of posterior circulation stroke that is used as a stroke screening and not severity, and was used uniformily on all patients. This is part of the baseline algorithm and was not the focus of our study.

The majority of diverted patients in our study had a LAMS of either 4 or 5, with 28 and 46%, respectively. All of the LVO patients had LAMS 4 or 5. Although our protocol defined selected patients for rerouting as those with LAMS 4–5, 26% of rerouted patients had a LAMS score <4. One reason these patients may have been rerouted is concern for major stroke despite low LAMS, possibly due to decreased level of consciousness. Further studies are needed to test other scales and selection methods for routing protocols.

Our 30 min allowance for travel to the Thrombectomy Center, did not negatively affect patient's functional outcomes or increase complications. In this “real world practice” observational study, we found that only 3% of the 303 patients who were diverted from a PSC took greater than 30 min to arrive at the Thrombectomy Center. This is notable because EMS estimates of transport time can be difficult in an urban setting where traffic patterns can be unpredictable. The use of apps with real-time traffic data may provide an advantage but needs to be studied.

A major concern about bypassing a PSC is that patients may then arrive outside of the IVT window. Our study showed that no patients who were transported within the time window for IVT became ineligible for IV therapy after being diverted to the nearest Thrombectomy Center.

Our study revealed a significant decrease in the LKW to groin puncture time for patients that were rerouted, compared with those that were transferred from other hospitals for thrombectomy, the median time difference was almost 2 h favoring rerouting.

While a greater proportion of rerouted patients treated with MT had a favorable outcome compared with patients transferred from other hospitals, this was not statistically significant. Other comparisons in our study did not meet statistical significance. This may be due to our low sample size and will need to be evaluated in a larger study. Our findings support those of multiple prior retrospective studies showing greater treatment delays in patients transferred between hospitals vs. those admitted directly to a Thrombectomy Center (28–32).

A concern with a rerouting protocol is potentially diverting patients away from a nearer hospital who do not require the resources of the Thrombectomy Centers and would take scarce beds needed for patients with tertiary needs. In our study, 15% of rerouted patients had ICH. Patients with ICH may benefit from being directly transported to a CSC, as there is some evidence that interhospital transfers of patients with ICH may be associated with worse outcomes (33). In a study on field validation of the LAMS, ICH patients were placed into the category of “CSC-appropriate” transports. The study group recognized that ICH patients were likely to benefit from access to faster reversal of anticoagulation, advanced neurosurgical, neuroendovascular, and neurocritical care capabilities that are offered at a CSC, and acknowledged delays that can occur with inter-hospital transfers. They found that LAMS of 4 or greater increased the likelihood that a patient had an underlying CSC-appropriate lesion, either ICH or ischemic stroke caused by LVO, by 2.5-fold (21). However, we must emphasize that our study did not seek to prove that rerouting ICH patients is beneficial and that this is an area of investigation that requires further studies. We also found that 35% of rerouted patients were stroke mimics, most of which (59%) were patients with mental status changes, intoxication/medication, and hypertensive urgency.

A significant limitation of our study was that it was conducted in an urban area with multiple Thrombectomy Centers within a 30 min range, which limits generalizability to regions with a relative dearth of such centers. As mentioned above, simulations suggest that even longer transport times could still be beneficial for patients in rural areas, so future studies looking at this specifically will be helpful. The protocol did lead to diverting a number of patients who were not eligible for any intervention, however many of these patients likely benefited from availability of a higher level of care offered by a tertiary center. The impact of re-routing on these patients outcomes should be be assessed in future research, however it was not the scope of this study.

In conclusion, implementation of a protocol that uses an EMS-applied clinical scale to select patients with stroke symptoms that would benefit from transfer directly to a Thrombectomy Center is feasible and did not adversely affect outcomes of stroke patients, resulting in significant faster times from LKW to MT. In our study outcomes of patients that underwent MT were better than patients that were first taken to a PSC and then transferred for treatment, however the difference did not reach statistical significance. The rerouting protocol did not result in missed opportunities for IVT.

## Data Availability Statement

The raw data supporting the conclusions of this article will be made available by the authors, without undue reservation.

## Ethics Statement

The studies involving human participants were reviewed and approved by Johns Hopkins University School of Medicine. Written informed consent for participation was not required for this study in accordance with the national legislation and the institutional requirements.

## Author Contributions

VU, EM, AG, MP, and AA contributed to conception and design of the study. JB, BJ, LT, KY, CS, and EL organized the database. MP performed the statistical analysis. TH wrote the first draft of the manuscript. VU, KAP, BT, and MP wrote sections of the manuscript. All authors contributed to manuscript revision, read, and approved the submitted version.

## Conflict of Interest

VU received funding from Genentech Inc for two unrelated studies. As site PI for the TIMELESS trial and as PI of the OPTIMISTmain trial. The remaining authors declare that the research was conducted in the absence of any commercial or financial relationships that could be construed as a potential conflict of interest.

## Publisher's Note

All claims expressed in this article are solely those of the authors and do not necessarily represent those of their affiliated organizations, or those of the publisher, the editors and the reviewers. Any product that may be evaluated in this article, or claim that may be made by its manufacturer, is not guaranteed or endorsed by the publisher.

## Abbreviations

LVO: Large Vessel Occlusion
LKW: Last Known Well
IVT: Intravenous Thrombolysis
MT: Mechanical thrombectomy
ICH: Intracranial Hemorrhage
SAH: Subarachnoid Hemorrhage
LAMS: Los Angeles Motor Scale.
